# A System of Physiatrics, or of Hippocratic Medicine

**Published:** 1837-07

**Authors:** 


### Art. VIII.

*System der Physiatrik, oder der Hippokratischen Medicin.* Von Ferdinand Jahn. *Erster Band. Physiologie der Krankheit und des Heilungsprocesses, oder allgemeine Pathologie und Jatreusiologie.* —*Eisenach*, 1836. 8vo. pp. 582.

*A System of Physiatrics, or of Hippocratic Medicine.* By Ferdinand Jahn. *First Volume. Physiology of Disease and Restoration, or General Pathology and Iatreusiology.*—*Eisenach*, 1835. 8vo.

In our last Number, in noticing the works of Professor Mayer and Dr. Mühry, we took occasion to remark briefly, but with some severity, on one or two of those deviations from the path of sound reason and philosophy to which, we are sorry to say, the physicians and physiologists of Germany are so prone. On the present occasion we purpose to lay before our readers, at considerably greater length, an account of a new system of Pathology which has lately been promulgated in that country, and which is said to have attracted many admirers, and not a few converts, among the younger members of the medical profession.

To some of our readers it may, perhaps, appear scarcely necessary to devote so much of our space to a work like the present; more particularly after seeing the judgment which a careful analysis may lead us to pronounce upon it. But, setting aside the amusement which we think our account of Dr. Jahn's book cannot fail to yield to many; as we feel it to be our duty to make our readers acquainted with every novelty of importance in medical science, whether of fact or opinion, we could not dismiss, with a mere passing notice, a publication which is the production of a man of undoubted learning and great talents, and which claims to be considered as the Koran of a new creed in the world of medicine. We are, moreover, well pleased to let our friends in Germany see that, while they can nowhere count on more cordial or sincerer admirers than ourselves, so long as they present us with works of sterling merit, they must not expect that productions like that now before us, which, under the garb of philosophy, set all true philosophy at defiance, can ever find favour in our sight.

It is a singular feature in the German character, that the same minds which are capable of undergoing the greatest drudgery for acquiring the knowledge of facts should be prone to indulge in the most extravagant fancies and most baseless speculations. Hence it is quite as frequently in the writings of the old as of the young, in the speculations of men of undoubted learning as in those of the novice and pretender, that we are constantly startled with the sudden intrusion of the most fantastic and visionary forms, altogether at variance, as we conceive, with the character of the author and the book. In this respect the follies of the Germans are unlike those of many other people: they are grave, learned, and elaborate, yet still follies. We have also our fools, and some who write books; but, however we may be led to laugh at their folly, we are never astounded by their learning.

The author of the work before us is evidently a very remarkable man. He answers but little to our idea of a modern physician, and would have been more at home, one would think, wandering over Europe by the side of the "god-like" Paracelsus, whom he is so fond of quoting, than confined in the little duchy of Meiningen, which, to such a restless spirit, can be little better than a prison. There is mystery in his book, but it is of a sacred, and not of a vulgar description. Though his system may be extravagant, he is evidently deeply in earnest, and therefore entitled to our respect; and his talents, however we may lament their application, are well calculated to arrest the attention of the readers of his works, in other countries as well as his own. Being of the school of Schelling, Dr. Jahn, of course, neglects the boundaries by which former philosophers divided science from science, and walks along, careless of technical prescription, holding high commune with universal nature.

His diction bears a stamp of religious solemnity, as if he had just left the presence of some mysterious power. We can really hardly imagine that he plays a simple part in that simplest of all dramas, German domestic life. He tells us that he has declined several offers of promotion to professorships in different universities. We do not wonder at it. No formally prescribed line of duty can possibly suit an individual who, it would almost appear, lays claim to inspiration. We can imagine him teaching at a porch, or healing by the wayside, or musing in solitude; but, with circumstances of a more modern state, or commoner kind, we find it impossible to invest him. Before we recount them, we must say something to our readers in extenuation of our author's extravagancies. His theories look quite different things in their German to what they will do in our English dress. We know not what is the reason of the fact, but it is one, that, in the country beyond the Rhine, many beautiful theories enjoy the sunny life of popular favour, which would scarcely survive their birth in our island. Translators have long discovered this truth; and we ourselves must confess that, in our German travels, we have often fallen in love with beautiful ideas, which we would fain have transplanted to British ground, but they would never have borne the change. Our intellectual climate is too severe for the fruits of German speculation.

Another work, or what may be regarded as the second volume of the present, has since appeared, written on subjects of a practical nature. This we shall notice hereafter; and, if our readers can believe, firstly, that the result of Dr. Jahn's treatment has been so successful as he states, and, secondly, that his practice is, as he says, founded on the principles detailed in the present volume, they may feel disposed to regard his theories with an eye of greater favour than they might seem entitled to for their own sake. He says, that, in the year 1831, of 293 cases treated in his hospital, where no trifling ones are admitted, he did not lose one; in 1832, out of 531, he only lost six,—viz. two from phthisis, one from dropsy and phthisis, one from a tumour in the cavity of the thorax, and one from catalepsy; in 1833, out of 533 cases, he only lost five! We may mention that this practical work is now to be obtained with difficulty, having been forcibly suppressed by the government, on account of certain observations, thought too bold, made in it respecting the condition of the peasantry and the state of society in the author's little state.

Dr. Jahn's pathology is based upon the physiological position that man is a kind of *recapitulation* of the animal kingdom,—i. e. that all the powers and functions of animal life are found in man, each in a degree of development consistent with the greatest possible perfection of the whole. Parts of the animal system may be more completely developed in the lower animals, but the whole is only perfect in man. Thus, the lymphatic system is more complete in certain fish than in the human economy, in which its development is impeded by the operation of more important systems. Each of these systems is capable of unlimited development; each strives to become predominant, and each is only kept in healthy limits by the counteracting influence of the rest. Now disease, according to Dr. Jahn, is the anormal predominance of one of the systems of animal life. When the arterial system developes itself to a degree of predominance inconsistent with the harmony of the whole organism, the result is inflammatory fever. A similar state of the venous system is manifested by venous congestion, and of the lymphatic, by scrofula. Seeing that, in these diseases, certain systems strive to become predominant, and usurp a disproportionate share of the vital powers, the consequent disease must represent the vital process of that class of animals in which such system is normally predominant. The carrying out of this position is the subject-matter of the book before us. The process of inflammation is declared to be that state, anormal in the human subject which is normal in the bird; scrofula is compared to the life of a fish, &c. &c.

It is not attempted by Dr. Jahn to prove the actual identity of diseases with the analogous normal conditions of animals; but this has been maintained by Dr. Carl Hoffmann, whose work has appeared since the publication of the *Physiatrik.* This conclusion of identification is one, however, to which there is no reason for asserting that our author has not arrived, though he generally contents himself with the more modest process of parallelizing.

In the following pages our object is, in the first place, to develop as much of our author's system as our limits will allow us; and, in order that our readers may have it presented to them as pure and as complete as possible, we shall reserve our criticism on it until the close of the article.

Every organism, says Dr. Jahn, has two bearings, an universal and an individual; *universal,* inasmuch as it proceeds from the great whole of nature, and tends inevitably to return to it again; *individual,* inasmuch as it strives, at the same time, to attain as high a degree of individuality as possible, and to form of itself an independent whole.

In the animal organism there are two grand systems: the *reproductive* or *vegetative,* and the *sensitive.* There are three processes of the former system which correspond to its *individual* direction,—i. e. which tend to make it a whole of itself, and remove it from the operation of the laws of universal nature: these three processes are, 1, the formation of lymph; 2, the formation and circulation of red blood; and 3, the conversion of red blood into animal fibre. There are also three processes which correspond to its *universal* direction,—i. e. which express its inevitable tendency to return to the whole from which it originally sprung, viz. 1, the interstitial absorption of animal tissue; 2, the conversion of arterial into venous blood; 3, the conversion of venous blood into excretory products. The character of the former direction of the vegetative system may be expressed by the words *ingestion* and *concrescence;* of the latter, by *liquescence* and *egestion.*

In the sensitive system, these conflicting directions are manifested by the states of action or tension, and of repose or relaxation. In the former conditions, the animal organism stands out as something separate and individual; in the latter, it manifests a tendency to merge in surrounding nature. The processes which correspond to the *individual* direction of the sensitive system are the following:—1. Action of the nerves of motion; 2. Perception by the nerves of sense and of sensation; 3. Perception by the ganglionic system; 4. Instinctive desire; 5. Action of the reasoning powers; and 6. Action of the will. The states which indicate the *universal* direction of the sensitive system are those in which the above actions, or processes, are succeeded by intervals of repose, in which the sensitive powers are in a state of abeyance.

Health consists in the harmonious counteraction of one of these directions by the other, and disease may be caused by excess in either.

Whenever a vital process which corresponds to the individual direction of the organism is developed to such an extent that, instead of being a part of a whole, it has become a whole of itself, the harmony of the system is irreparably disturbed, and death is the consequence. The same result occurs whenever the universal direction predominates at the expense of the individual.

The proper classification of diseases therefore must, according to Dr. Jahn, correspond to the vital processes which we have enumerated. We commence with the vegetative system, and with the formation of lymph, its first process, of which the anormal development constitutes *scrofula.* In this disease the lymphatic system endeavours to establish itself independently, and to become predominant in the general organism, trying, as it were, to subvert the law by virtue of which it is a part of a whole. It resists the conversion of its lymph into arterial blood. Hence hypertrophy, swelling, and congestion of the lymphatic vessels; the whole of which, instead of being subordinate, begin to be governed by their own laws, to establish their own centres, and to resist foreign influence. In the degree that this system is predominantly developed, the other vital processes, and particularly those of arterialization and nutrition, to which it is its principal province to minister, are of course diminished. In short, the life of the whole organism, which only flourishes in the degree in which harmony prevails amongst the systems which compose it, is prostrate.

The next process of vegetative life is the formation and circulation of arterial blood, which, when anormally developed, constitutes arterial plethora, simple fever, synocha, &c. In these affections the red blood becomes the most prominent agent in the system, and its tenacity in retaining its characteristic qualities indicates its tendency to independence. The pulse is full and strong, the complexion redder, the temperature higher, the blood drawn from the veins is of a deep red colour, and coagulates quickly and firmly. The heart and larger vessels are more developed, and actually increase in size. (Schoenlein.) In proportion to the morbid extent of the process of arterialization is the state of depression of the other processes of vegetative and sensitive life. Muscular and nervous activity are prostrate, the sphere of mental operation diminished; reproduction, excretion, and secretion arrested altogether, or imperfectly executed. Inflammation of a viscus, or of any part of the system, is local excess of arterial life. In the organ affected, the globules of blood resist the metamorphosis into the ordinary molecular elements of animal tissue, and are incorporated with the latter without undergoing any essential change, so as to give it the red appearance which is characteristic of inflammation. They are equally obstinate in resisting conversion into secretory and excretory products. Thus, the general organism is no longer united and harmonious; a kind of *status in statu* arises, and the arterial system, instead of being subservient, as formerly, becomes independent, and consequently diseased. When the inflamed vessels succeed in withdrawing themselves from the operation of the laws by which the system at large is governed, either suppuration or gangrene is the consequence. The former is a reduction of the part into elements of lower vitality, of an infusorial nature, (Treviranus;) the latter into its chemical constituents.

The third process of the vegetative system is the formation of animal tissue from the globules of the blood. This process, when anormally developed, constitutes hypertrophy. Its converse, atrophy, is caused by excess of interstitial absorption. When the fifth process of vegetative life is developed to too great an extent, we have venous congestion. Here the veins display the same tendencies as the arteries in arterial plethora. The venous system usurps the greater part of the vital powers, and debilitates, in consequence, the functions of other parts of the body. The blood which it contains no longer complies with the law of the system, by virtue of which it ought to be converted (in the liver, for instance,) into excretory products, but remains in its original condition in the portal veins. The general congestion prevents the interstitial absorption, of which the veins are the principal agents, from properly taking place. Finally, the latter engross a disproportionate quantity of blood, so that the arteries suffer a defect both in the quality and quantity of their contents. Typhus indicates a state of the venous system similar to that of the arterial in synocha. Profuse excretions are caused by the excess of the sixth process of the vegetative system.

We now come to the processes of the *sensitive system.* When the first of these which we have enumerated,—viz. the action of the nerves of motion,—withdraws itself from the operation of the laws of the general organism, and is called into independent play, convulsions are the consequence. As in local inflammation, the vessels affected are no longer under the control of the heart, or of the laws which govern the arterial system generally; so, in convulsions, the state of the nerves affected is similar with regard to the nervous centres. An anormal degree of perception of the nerves of sense and of sensation is denominated *algia.* When a similar state obtains in the ganglionic system, singular phenomena are the result. Their aggregate is usually denominated *somnambulism* or *mesmerism.* In the normal state of the human subject, perception by the ganglionic system, which is so developed in lower animals, is superseded by the cerebral functions. But, in the anormal condition of which we are speaking, its exercise would appear, even in the human subject, to predominate over the latter; for, during somnambulism, the intellectual faculties, and the higher powers of life, are temporarily suppressed. In their place, we observe the instinctive apprehension, presentiment, and foreknowledge, which we are accustomed to admire in lower animals, particularly in insects. "In the same way that the queen-bee clearly perceives the gender of the young which she still carries in her womb, and acts accordingly as to the size and construction of the cell to which she is to consign it;—in the same way that the working bees anticipate the wants of the brood which is yet unborn, and that instinct, generally, whether exercised for the progeny of animals or for their own preservation, overlooks both time and space,—so are human beings, in whom perception by the ganglionic system is anormally developed, capable of perceiving the internal condition of their own or of other bodies, of discerning whatever has relation to themselves, and of extending their mental vision to a distant time or place."*

Instinctive desire is also liable to the excess which we have described

* Schubert.

in the other functions, as constituting their respective diseases. Hence *satyriasis*, *nymphomania*, *polyphagia*, &c.

That state of the reasoning powers in which they have lost their healthy relation to the general organism,—their normal connexion with the organs of sense, the will, the impulses, and voluntary motion,—is denominated *madness*. Mania is caused by an independent and predominant action of the will, which is thus removed from the influence of the reasoning powers, and appears to be exercised for its own gratification. Of course, a contrary condition of these several functions may make its appearance; such a state constitutes paralysis of the nerves of motion, and anæsthesia of those of sensation.

Such is our author's classification of diseases. The following is the account he gives of their nature and bearings.

The vital processes which we have enumerated are more or less common to the whole range of organized beings. Some are more prominent in one class, and some in another. Man, who is at the head of the animal creation, presents them in the greatest degree of aggregate perfection. Now, when one of these processes is anormally developed in the human organism, the phenomena to which this anormal development gives rise must necessarily resemble the healthy functions of the lower organism in which such process is naturally predominant. This doctrine is alluded to in Plato, and has been adopted for the explanation of mental diseases by Nasse, of malformations by Meckel, and of other affections by Carus, Oken, &c. In pursuing this analogy, we shall follow, of course, the classification of diseases which we have adopted above.

The general condition induced by scrofula presents numerous points of similarity with the healthy organization of cartilaginous fish, in which the lymphatic and mucous systems naturally predominate. In these animals, as in the disease under consideration, the blood contains little fibrine; the muscles and bones are imperfectly developed, the former being pale, the latter more or less cartilaginous; the cavity of the thorax is contracted; the organs of respiration retarded in their growth; the abdomen, intestines, and liver, on the contrary, of considerable size. *Gluttony is equally characteristic of the disease and of the animal!* Other points of analogy are supplied by the paleness of the blood of scrofulous subjects, and by the varicose dilatations of their lymphatic vessels.

In respect of predominant arterialization, the anormal phenomena which synocha produces in the human subject are strikingly analogous to those presented by ravenous animals in a healthy state. (Stark.) Witness the full, strong, and rapid pulse; the reddened, fiery, shining, and wild-looking eye; the red and rough tongue; the limited secretion of urine, its high colour, strong odour, and acidity; the hard and dry fæces; the hot and rapid breath; the increased temperature of the blood; the extraordinary muscular power; the great restlessness; and even the *delirium furiosum*. In inflammation, the human subject displays the blood-heat and intensity of respiration of a bird of prey. (Kieser.) Even the common consequences of inflammation,—the adhesion of serous membranes with contiguous parts, &c.—are normal conditions in several animals!

The hypertrophy, which is morbid in the human subject, is natural in some lower organization. Enlarged liver has its prototype in the Molluscæ; enlarged salivary glands, in the Ruminantia; the hypertrophied heart, in the bird; aneurism, in different fish; of the aorta, for instance, in the dolphin, sea-dog, &c. (Meckel, Carus, Albers, &c.;) sacculous distention of the œsophagus, in numerous birds; hypertrophied muscular coat of the stomach, in the horse; and distended stomach, in the leech. Local depositions of fat, which constitute diseases in the human subject, are normal in the camel. Emphysema is presented by numerous lower animals in their healthy condition, (Carus,) &c. &c.

Predominant vitality of the venous system is a normal state in living and hybernating animals. During the sleep of the latter, respiration is very imperfect, and the blood of the veins and arteries nearly similar in colour and quality. Venous vitality is prominent in fish, to whose healthy constitution the state of the human subject in chlorosis is strictly analogous. Witness, in both cases, the thin, pale, and watery blood; the laborious respiration, the diminished temperature; the infiltration of the cellular tissue with yellowish, tenacious, half-coagulated matter; the inflated appearance of the body, &c. &c. Morbid products, which owe their origin to venous congestion, and amongst which are to be classed the greater part of the urinary calculi, have their prototypes in numerous animals. In the hog, we find ossification of the substance of the heart; in the tortoise, there are small bones between the arteries rising from it; and in the sturgeon, ossification of the aorta, &c. &c.

We now pass to the diseases corresponding to the functions of the *sensitive system.*

Convulsions may be compared to healthy movements of a similar kind, which we observe in several lower animals. Anormal sensibility has also its prototype in inferior organization. Of excessive perception by the ganglionic system, we have already sufficiently spoken. Predominance of instinctive desire is identified, even by the vulgar, with the propensities of lower animals. In the different forms of madness, the human being betrays moral qualities which bear a greater or less similarity to those characteristic of different animals. In mania, and in the natural constitution of carnivorous animals, there are alike observable increased muscular power, perpetual restlessness, nocturnal excitement, violent agitation, a glaring eye, and destructive propensities. Elephants and similar animals, in their thoughtful manner, tenacious attachments, obstinate antipathies, and in their disposition to indulge in revenge, manifest many of the qualities which characterize the human melancholic. The imbecile resembles the ruminating species in the obtuseness and stupidity of his nature, and in the trifling extent of his intellectual powers. Almost all our moral diseases find their normal prototypes in inferior organizations. The propensity to murder, thieve, and destroy is implanted in numerous animals. In others we find a natural melancholy, a shyness of their fellows, and an intolerance of all intrusion; in others, again, the greatest restlessness and irritability; and, finally, in some qualities which are comparable to those of an impertinent fool!

As to the converse states of the sensitive functions,—i. e. those in which their activity is diminished, instead of increased,—it is unnecessary for us to pursue an analogy with lower organizations, which must present itself at once to every mind. In descending the scale of organized beings, we find the sensitive system become more and more defective, till it finally ceases in the vegetable kingdom.

But all diseases are not confined to processes which find their prototype in a lower animal organization: there are numerous affections of the human subject which are to be compared with phenomena of the vegetable, and even of the mineral kingdom. In diabetes, we have the production of saccharine matter, so richly supplied by numerous plants. Concretions of phosphate of lime are found in several vegetables. Benzoic acid, which is common in the vegetable kingdom, is often secreted in diseases of the kidneys. Collections of air, termed emphysema in the human subject, are frequently met with in vegetable structures. In many plants, dropsy is a healthy condition. (Andrews.) Frank has already compared lymphatic and coagulable transpirations in the human subject with the gummy secretions of trees in a fertile soil. The section of a carcinomatous excrescence bears a remarkable resemblance to that of a vegetable, and its manner of growth is similar. Stark describes a wart as being strictly analogous to a plant, &c.

Examples of *chemical* processes taking place in an animal organism are furnished by the formation of stony substances, by the morbid elimination and combination of combustible gasses, by the anormal play of electrical agents, by melanosis, &c.

Inasmuch as the disease of one organization is constituted by its assuming the character of another, it is possible, continues our author, for the human female, who, according to the latest researches, is an imperfectly developed male, (Walther, Wagner, and Tiedeman,) to simulate, in a diseased state, the constitution of the latter. That the male may fall back to a more or less feminine organization results from the preceding observations; and facts are not wanting to warrant the conclusion,—e. g. the *morbus mulieris*, whose home was formerly the ancient Scythia, but which has lately been found in other regions, (Larrey;) the gradual disappearance of the male organs of generation, and the secretion of milk by males; a phenomenon of frequent occurrence amongst the Mongolians and South American tribes.

Notwithstanding all that has been stated, inasmuch as the morbid process which constitutes disease is still necessarily, more or less, under the influence of the general laws of the organism, it follows that its products can obtain but a very imperfect degree of organization. Hence all excrescences and pseudo-formations resemble the lowest animal and vegetable forms. Should they simulate the higher, it is in an extremely imperfect and fantastic manner. Their origin resembles that of the lowest organized beings: they first exist as simple, homogeneous, formative tissue; subsequently, this divides into globular portions, the peripheries of which coagulate, and form coverings for the centres, which either remain more or less fluid, or undergo an organic crystallization. This is the way in which hydatids and tubercles are produced. Should traces of a sanguineous system be discovered in these morbid organizations, they will never be found to advance further than the first rudiments of vascularity. The hair, teeth, and bones sometimes found in membranous sacs, are instances of the imperfect and fantastic imitations of higher organization mentioned above. Even the morbid products which display the most perfect organization and most complete individuality, merely simulate the character and type of the lowest animals and plants; the *entozoa* correspond either to *infusoria, polypi or annularia,* and the vegetable formation to the most imperfect plants. It is a law of animal and vegetable organisms of a low grade, that, at an advanced age, they soften and melt. This obtains of sponges *medusæ* and various *fungi.* Morbid organizations are also harder at an early than at a late period of their existence, and are destroyed by softening down at their centre, and by a gradual conversion into fluid matter, &c.

We pass to the consideration of the different types which diseases assume.

Every vital process may be said to intermit, to be made up of impulses and pauses, and to oscillate between action and repose, between tension and relaxation. The same intermittent tendency is observed throughout the operations of nature. In the animal organism, this intermittence is expressed by the states of action and of repose, of sleep, and of wakefulness. Moreover, it is not only manifested by the system, taken collectively, but by each of the functions which compose it. Now, as disease is nothing but an immoderate development of a process of the healthy organism, it is plain that the type which it assumes must correspond, more or less, to that of the latter.

The period and extent of intermission are alike various in the vitality of lower animals and in the diseases of the human subject. Fish never sleep; several vegetables are perfectly alive in winter; and some of the hybernating tribes are much more apathetic during that season than others. Again, the vitality of the organs of sense intermits much more distinctly than that of the secreting organs, the cellular tissue, &c. Thus, also, there are diseases which appear entirely to cease for a day or more, and others which continue their career with an almost imperceptible intermission. That some are without a type,—i. e. without certain intervals of diminished intensity,—may be a doctrine of the schools, but is not a pathological truth. The period to which intermission of vital action may extend in different animals is extremely various. Some of the hybernating tribes sleep two, and some six months, in winter; the natural sleep of some animals only lasts two hours, that of others twelve. In like manner, fits of hooping-cough occur at intervals of an hour or more; of intermittent fever, at an interval of one or more days; whilst months intervene between the regular exacerbations of syphilis and dropsy, &c.

Our author next considers the stages into which the course of a disease is to be divided.

All life (he says,) is a process of development; no organization manifests the same perfection, no vital power the same intensity, at the commencement and at the termination of its existence. Life gradually attains a culminating point at which it cannot remain, but whence it must gradually descend. Thus it has a period of evolution, or of development and growth, of culmination or of perfection, and of involution or of descent and decay. Man, to use the words of Reil, commences as a bright point, becomes a sun which governs the world, and finishes as a spark in the waste of night. Now, inasmuch as every disease is a vital process, tending to establish, or actually establishing, a separate organization, (*status in statu,*) it must necessarily go through the stages which are common to every organism. We shall find, too, other analogies between diseases and vital processes, besides the mere fact of their both running through certain periods of development and decay. Many organic beings undergo metamorphoses during their different stages. In the same way, diseases undergo organic metamorphoses; so that, in an advanced stage, they may become quite dissimilar to what they were in a former. Syphilis passes in its career through all kinds of forms; leprosy quite changes its appearance as it proceeds; tubercles undergo a complete transformation.

In the same way that beans will germinate after a lapse of two hundred years, and onions after two thousand, that the spawn of fish and the eggs of many insects may lie for a long period without manifesting a trace of vitality, so can the germs of numerous diseases remain latent for years.

Again, there are parasitical diseases corresponding to parasitical animals and plants. Hospital gangrene, many forms of chronic dropsy, petechiæ, &c., are, as it were, parasitically inosculated upon other diseases, in the same way that numerous fungi, sponges, mosses, and worms, live exclusively at the expense of a foreign organization.

Some plants act prejudicially, and some beneficially, upon the growth and health of others. The *ervum hirsutum* and *ranunculus arvensis* only flourish amongst corn. On the other hand, oats never prosper in the neighbourhood of the *serratula arvensis;* nor wheat in that of *erigeron acre;* not hemp near the *euphorbia peplus.* In like manner, syphilis increases in intensity when prevailing in a subject afflicted at the same time with scrofula, scurvy, or the itch. On the other hand, the cow-pox and variola counteract each other, and the same antagonistic relation obtains between some other diseases.

Numerous animal and vegetable organisms alter their general character in changing climate or country. Our *æthusa cynapium* loses its disagreeable odour when transplanted to America. Change of climate converts trees into bushes, annual plants into perennial, &c. The same fish may present a different colour and shape in different parts of the ocean. In Corsica, animals are chiefly speckled and party-coloured; in Syria, almost all animals have long white hair; in Cubagua, pigs degenerate into a species with claws half a span long; in Lapland, the dog is small and ugly; in the south, handsome and well made. The human form is equally subject to the influence of climate. Diseases, too, assume different forms in different countries. Syphilis is not the same in Scotland as in Egypt, in Peru as in North Asia, in Scandinavia as in Italy. The same obtains of the itch, to which physicians have given a new name in every new climate in which they have met with it.

There are substances which act specifically upon certain organisms. Camphor is poisonous to insects, pepper to fowls, and quassia to flies. Buttermilk produces a fatal colic in the horse, an animal which can swallow pounds of belladonna with impunity. Diseases also have their poisons, their specifics. Amongst these are to be reckoned mercury for syphilis, sulphur for the itch, bark for the ague, &c.

The phenomena which accompany the abortion of the animal embryo are the same which are induced in order to stifle the germs of disease. The former may be brought about by emetics or purgatives, which produce a shock throughout the frame of the mother, or by emmenagogues, which stimulate to greater secretion the organ where the fœtus is developed. The same emetics and purgatives frequently annihilate the germs of disease, &c.

All organisms have their origin in generation: to this rule there are no exceptions; and it must, therefore, hold good of diseases, which are morbid organisms, whether they are produced by contagion, or whether they arise spontaneously. In the latter case, the external deleterious agents represent the male principle, the actions of the recipient matter, the female; the common product is the disease!

In every process of generation, the constitution of the matrix influences, to a great extent, the character of the product. The operation of the same external agents does not produce the same kind of infusoria in mucus as in pus, (Gruithuisen;) animal matter in the first stage of its decomposition is resolved into large, later into small, infusoria, (Treviranus;) the infusoria furnished by an infusion of bruised seeds are smaller and shorter lived than those of an infusion of unbruised, (Spallanzani;) *entozoa* differ in form and construction according to the manner of life, age, and part, of the individual in whom they are developed. Diseases, also, vary according to the state of the subject in whom they are produced, the matrix in which they are engendered. Those of plants differ from those of the lower animals; those of man from both; and the latter are diverse according to the peculiarities of the individual, or the part of the system, affected.

But, in the generation of infusoria, the qualities of the external agents, as well as those of the matrix, affect the nature of the product. Their form and structure varying according to the intensity of light, shade, heat, &c. to which the matrix is submitted, according to the shape of the glasses in which it is contained, to the nature of the air by which it is surrounded, &c. The same obtains of external agents engendering disease. The air of a prison and a cold north-east wind produce different diseases in the same individual. The operation of every poison is peculiar to, and therefore characteristic of, itself.

In the same way that only lower animal and vegetable organisms originate in spontaneous generation, and that all higher ones are produced by the development of seed, so it is only the more imperfect diseases which are spontaneous, whilst all the better marked and independent ones, such as small-pox, the plague, syphilis, &c. are engendered by contagious matter; &c. &c.

But here our limits compel us to take leave of Dr. Jahn, and of his endless series of analogies. We have given, however, quite sufficient to shew the spirit in which his singular book is written; and more than enough, we suspect, to satisfy the curiosity of most of our readers. In taking leave of it, we will only say that, although Dr. Jahn and some of his countrymen may probably set it down to some peculiar obtuseness of comprehension on our part, we must here confess our want of admiration for the ingenious analogies of which it is for the most part made up. Without saying that we regard such ingenuities with contempt, we certainly look upon their construction as a waste of time, and a misapplication of faculties given for better purposes. Excusable in a very young man, prone to acknowledge resemblances, they shame maturer age, when the attention should embrace a wider range of qualities, and discern differences. The exercised reason should, we think, eschew the loose similitudes whereupon our German friend bases his assertion, that an enlarged liver has its prototype in the molluscous animals. It betokens, in our opinion, a most incomplete and unphilosophical consideration of the peculiar endowments of woman to libel the human female (and her Maker,) by proclaiming her an imperfectly developed male. There is nothing logical or satisfactory in the assertion that, because diseases are organisms, and organisms are only produced by generation, therefore diseases are produced by generation; and therefore external agents are the male principle, the recipient matter the female, and the disease the veritable child. Even the facts adduced in support of Jahn's analogies are often doubtful; and of the theory—it is surely "of imagination all compact." We may even add, that his views have not the merit of novelty or invention, being little more than cast-off follies dressed up in the finery of new phrases; and, as it were, a remodelling of "the ancient opinion" alluded to by Bacon, "that man was *microcosmus*, an abstract or model of the world," and which he speaks of as having been "fantastically strained by Paracelsus and the alchemists, as if there were to be found in man's body certain correspondences and parallels, which should have respect to all varieties of things, as stars, planets, minerals, which are extant in the great world." This manifest foreknowledge of Jahn's darling theory may, perhaps, not without advantage, induce him to look into the chapter of the great philosopher wherein he speaks of the Three Distempers of Learning, whereof, we regret to say, certain physiologists and certain botanists in the present day are grievously affected with at least one; "fantastical learning, or vain imaginations."

---

